# From compute to care: Lessons learned from deploying an early warning system into clinical practice

**DOI:** 10.3389/fdgth.2022.932123

**Published:** 2022-09-05

**Authors:** Chloé Pou-Prom, Joshua Murray, Sebnem Kuzulugil, Muhammad Mamdani, Amol A. Verma

**Affiliations:** ^1^ Data Science and Advanced Analytics, St. Michael's Hospital, Unity Health Toronto, Toronto, ON, Canada; ^2^ Department of Statistics, University of Toronto, Toronto, ON, Canada; ^3^ Department of Medicine, Faculty of Medicine, University of Toronto, Toronto, ON, Canada; ^4^ Institute of Health Policy, Management and Evaluation, University of Toronto, Toronto, ON, Canada; ^5^ Dalla Lana School of Public Health, University of Toronto, Toronto, ON, Canada; ^6^ Vector Institute, Toronto, ON, Canada; ^7^ Leslie Dan Faculty of Pharmacy, University of Toronto, Toronto, ON, Canada

**Keywords:** machine learning, healthcare, deployment, early warning system, clinical pathway

## Abstract

**Background:**

Deploying safe and effective machine learning models is essential to realize the promise of artificial intelligence for improved healthcare. Yet, there remains a large gap between the number of high-performing ML models trained on healthcare data and the actual *deployment* of these models. Here, we describe the deployment of CHARTwatch, an artificial intelligence-based early warning system designed to predict patient risk of clinical deterioration.

**Methods:**

We describe the end-to-end infrastructure that was developed to deploy CHARTwatch and outline the process from data extraction to communicating patient risk scores in real-time to physicians and nurses. We then describe the various challenges that were faced in deployment, including technical issues (e.g., unstable database connections), process-related challenges (e.g., changes in how a critical lab is measured), and challenges related to deploying a clinical system in the middle of a pandemic. We report various measures to quantify the success of the deployment: model performance, adherence to workflows, and infrastructure uptime/downtime. Ultimately, success is driven by end-user adoption and impact on relevant clinical outcomes. We assess our deployment process by evaluating how closely we followed existing guidance for good machine learning practice (GMLP) and identify gaps that are not addressed in this guidance.

**Results:**

The model demonstrated strong and consistent performance in real-time in the first 19 months after deployment (AUC 0.76) as in the silent deployment heldout test data (AUC 0.79). The infrastructure remained online for >99% of time in the first year of deployment. Our deployment adhered to all 10 aspects of GMLP guiding principles. Several steps were crucial for deployment but are not mentioned or are missing details in the GMLP principles, including the need for a silent testing period, the creation of robust downtime protocols, and the importance of end-user engagement. Evaluation for impacts on clinical outcomes and adherence to clinical protocols is underway.

**Conclusion:**

We deployed an artificial intelligence-based early warning system to predict clinical deterioration in hospital. Careful attention to data infrastructure, identifying problems in a silent testing period, close monitoring during deployment, and strong engagement with end-users were critical for successful deployment.

## Introduction

Despite advancements in machine learning algorithms for solving healthcare problems, there still remains a gap between the number of developed algorithms and the number of successful deployments (1, 2).

Problems can arise at any stage of deployment (3). Prior to model development, unclear problem definition is often cited as a barrier to successful deployment (4). Then, during model development, the training data can be biased, either due to missingness of vulnerable populations, small sample size, or erroneous data (5). When transitioning to production data, there can be a drop in model performance from test data to production data, which may result from changes in data formats, timing, and context (3). Problems can also arise with out-of-distribution generalization and incorrect feature attribution (for example, if clinical protocols or target populations change over time) (6). If a model makes it to the deployment phase, end-user engagement is a crucial facilitator of, or barrier to, successful uptake. Introducing a clinical team to a ML model may require changes in workflow and change management. Ensuring that end-users correctly use a deployed product is difficult if there is no buy-in or trust (7).

Because of the issues listed above, there are few successful deployments of ML in healthcare settings. The scarcity of deployments means there are no widely accepted “best practices” or standards by which to evaluate the success of a deployment. Recent work has looked at assessing the quality of the data that goes into model through the creation of “Datasheets for Datasets” (8). In the past year, Health Canada, the U.S. Food and Drug Administration (FDA), and the United Kingdom's Medicines and Healthcare products Regulatory Agency (MHRA) have released the Good Machine Learning Practice (GMLP) guiding principles, a document providing 10 principles to address deployment of healthcare algorithms (9). More recently, the DECIDE-AI steering committee have released DECIDE-AI, a set of guidelines and checklists meant for early live clinical evaluation.

In Fall 2020, we deployed CHARTwatch to the General Internal Medicine (GIM) ward at St. Michael's Hospital, an inner city teaching hospital in Canada (2, 10). The GIM ward currently holds 78 beds and receives approximately 4,000 admissions each year. Here, we describe in detail, the system's infrastructure and assess the success of our deployment through quantitative metrics (such as model performance, end-user engagement, and adherence to workflows) and by comparing our deployment to the GMLP principles. The purpose of this manuscript is to provide concrete insights into the deployment of ML in a healthcare setting and highlight opportunities to strengthen GMLP guidance.

## Materials and methods

### Model development

We developed a model to detect inpatient deterioration, defined as in-hospital death or transfer to the intensive care unit (ICU).

We obtained historical development data through the hospital's enterprise data warehouse. We used the following data sources: demographic information (sex and age), laboratory and vitals measurements. Our dataset consisted of all complete inpatient admissions to the GIM service between the dates of April 1, 2011 and December 11, 2019. We split the data into training and validation based on calendar date. Then, following silent deployment, we used data generated in the production environment between January 1, 2020 to May 30, 2020 as our test dataset. In the training and validation sets, we excluded any visits with length of stay less than 8 h or more than 40. The exclusion criteria were not applied to the test dataset. This was done to avoid biasing model development with outliers but to ensure accurate reporting of expected performance in a production environment. During training, death on the ward, transfer to the ICU, transfer to the step-up unit (a 4-bed unit on the GIM ward for higher acuity patients), and transfer to palliative care were used as the outcomes. Model performance was ultimately evaluated on the composite outcome of ICU transfer and in-hospital mortality.

We processed the data into a timeseries of 6-hour windows for each patient encounter, from admission timestamp to the first of either discharge timestamp or outcome timestamp. We took the mean value of numeric features (laboratory and vitals measurements) when the data were recorded multiple times within the same interval.

We observed a few data quality issues caused by data entry errors. For example, we found a body temperature value of 700 °C (see Table 1 for more examples of laboratory and vitals measurements). To address this, we processed all of our numeric features in the following way: we trimmed numeric features that were less than the 1st percentile or greater than the 99th percentile (as determined from the training data), and normalized the values to be between 0 and 1, using the 1st and 99th percentile. We then created “[feature]_measured” variables to indicate whether the feature was measured in the 6-hour window and “[feature]_time_since_last_measured” variables to keep track of the number of hours since the feature was previously measured. To address missingness, we imputed data with the last observation carried forward, followed by mean imputation. Details on data processing can be found in (10).

**Table 1:** Example of measured labs and vitals in the CHARTwatch training dataset. For these vitals and labs, we report their mean values, the 1st quantile (Q01), the 99th quantile (Q99), minimum value (Min) and maximum value (Max). The minimum and maximum values often fall outside of the range of biologically possible values (e.g., a maximum body temperature value of 6932 °C).

Feature	Mean	Q01	Q99	Min	Max
Vital—temperature	36.91	34.8	38.3	0	6932
Vital—diastolic blood pressure	71.49	47	101	0	173
Vital—systolic blood pressure	129.89	85	183	1	16,070
Vital—respirations	20.53	15	28	0	20,147
Lab—troponin	0.28	−1.9	5.58	−1.9	7.13
Lab—HBA1	−2.73	−3.14	−1.9	−3.3	2.29
Lab—glucose random	1.95	1.22	3.29	0	4.51
Lab—Hemoglobin	106.09	63	160	1	214
Lab—Basophils	0.03	0	0.13	0	2.69
Lab—Alanine Aminotransferease	3.38	1.61	7.08	1.61	8.78

Following numerous experiments with various machine learning methods, including logistic regression, Lasso regression, generalized additive models, and neural networks (10), we trained a “time-aware MARS model” to predict patient deterioration. This model consisted of two components: (1) The Multivariate Adaptive Regression Spline (MARS) used all processed input features to get a score of patient deterioration for each 6-hour window. (2) Then, additional features were created from the MARS scores (for example, current MARS score, baseline MARS score, change in score from previous time window, change since baseline) and were given as input to a logistic regression model. We selected this approach because it achieves similar performance to the more complex ML models, intrinsically incorporates a degree of feature selection, successfully models non-linear interactions, and was computationally efficient for deployment.

The risk scores generated by the time-aware MARS model were then categorized into “High risk”, “Medium risk”, and “Low risk”. We used 10-fold cross-validation on the validation set to pick the thresholds that would yield a visit-level positive predictive value (PPV) of 40% and a negative predictive value (NPV) of 99%. This threshold was selected because clinicians expressed the need to minimize false alerts, and they recommended a ratio of 2 false alerts to a single true positive (10). Models were not selected or adjusted after assessing performance in the validation dataset.

### Description of system

CHARTwatch was developed using the open source programming language R and the codebase was deployed to a local server with access to the hospital source systems. Various automated scripts are scheduled to run at different intervals. A summary of the scripts is provided in Appendix Table 1. The main CHARTwatch pipeline script runs hourly. It connects to the hospital databases, extracts the current patient census, and then pulls demographics, labs, and vitals data for current GIM patients. The script then does data cleaning, data processing, model prediction, and risk group assignment. The outputs of the CHARTwatch pipeline script are then communicated to clinicians through different methods, which are part of a comprehensive clinical intervention that was designed by an interdisciplinary team (the team has previously been described) (2):

- “High risk” alerts are sent in real-time to the GIM physicians through a paging application—“SPOK”^1^—running on the GIM team phones and the charge nurse phone. At our hospital, each GIM team and on-shift charge nurses carry a hospital-assigned mobile phone device 24-hours per day. Typically, the GIM team phones are carried by in-house residents.
- Patient risk groups are displayed, and updated hourly, in a locally-developed “electronic sign out” tool, which is used by GIM physicians to organize their teams.
- Emails are sent twice a day to the GIM charge nurses. This email contains the census of all GIM patients and their CHARTwatch risk group. The email is used by the charge nurses when they are assigning bedside nurses for the next 12-hour shift. They proactively attempt to match more experienced or skilled nurses with higher risk patients and to avoid one nurse from having multiple “High risk” patients.
- An email is sent once daily to the Palliative care team. This email contains a list of all patients who received their first “High risk” prediction in the past 24 h. The palliative care team contacts the patients' GIM physicians to ask whether a palliative care consultation would be helpful, with the goal of improving access to high quality end-of-life care for “High risk” patients, when appropriate.

“High risk” alerts to the mobile devices are triggered if a patient is classified as “High risk” by the CHARTwatch model. To minimize alert fatigue, the following alerting protocol is applied:

- After an alert, no further alerts are sent for the same patient for the next 48 h.
- Subsequent alerts only occur if a patient's status changes from “High risk” to “Low risk” or “Medium risk”, and then back to “High risk”.
- Alerts for patients who are transferred from the ICU to the GIM ward are silenced for 24 h after transfer, as these patients are already known to be “High risk” and are proactively followed by the critical care response team.
- In March 2022, in response to feedback that a small number of patients were getting a very large number of alerts, we began silencing all alerts after the fifth alert for a patient, although their status is still indicated as “High risk” in other communications.

A clinical pathway for “High risk” alerted patients was developed by an implementation team as described in detail elsewhere (2). This pathway was continuously refined by an implementation committee composed of GIM, ICU, and palliative care physicians and nurses, the chief medical resident, a clinical informatics specialist, and the lead data scientist. The committee met weekly through the pilot and early phases of the implementation and then was scaled back to meet monthly.

### Silent deployment and pilot phase

CHARTwatch silent deployment was affected by the onset of the COVID-19 pandemic. We initially planned a 4-month silent deployment, which was subsequently extended for 6 more months to accommodate the clinical changes that were being made amidst the challenges of the first wave of the pandemic. During silent testing, we used weekly check-in meetings with stakeholders to ensure the system was running smoothly and had some preliminary training sessions with end-users to assess buy-in and trust. The weekly check-in meetings included members from the following groups: the model development team, hospital Information Technology (IT), clinical informatics, and clinicians working on GIM, ICU and Palliative Care.

We began silent deployment in November 2019 and planned to launch the intervention in March 2020. During this time, we focused on several data-related issues. First, our testing period coincided with the hospital changing from traditional troponin measurement to a new “high sensitivity” troponin assay. In order to address this change, we modified our pre-processing code to scale the lab value accordingly. Earlier versions of CHARTwatch relied on medications and nurse notes. However, this silent testing period uncovered database connection issues, and these data sources were subsequently removed. This had no impact on model performance.

The onset of the COVID-19 pandemic resulted in numerous changes on the GIM unit, as this was the unit primarily responsible for care of COVID-19 ward patients. This resulted in physical changes to the ward, relocation of patients to other units in the hospital, and creation of new clinical care teams. All of these changes needed to be accommodated in CHARTwatch, including ensuring the model continued to identify the correct cohort of GIM patients and would be delivered to all the relevant care teams. We made a plan to deploy the model for GIM patients with COVID-19 as well as for those with other illnesses. Once these changes were made, we focused on model validation and data quality to ensure accurate performance (see Results section for details).

In August 2020, we initiated the pilot phase of our intervention. We began by deploying the system for 2 of the 7 GIM teams. Deployment progressed in a phased approach over a 6-week period, rolling out to all GIM teams and then to nurses and the palliative care team. The system was fully running by October 20, 2020.

The silent deployment and pilot phases were essential as it allowed the technical team to uncover issues with pipelines and workflows, and also allowed the clinical team to collect feedback from end-users.

A summary of major changes resulting from silent deployment and iterative refinement of the solution during the pilot and implementation phases is provided in Appendix Table 2.

### Downtime protocols

To ensure that CHARTwatch could run smoothly with minimal interruptions, all deployed scripts were developed so that emails to the project team would be sent out if any script failed. Furthermore, the data extract scripts were set to run hourly. If data extraction failed, the model outputs could rely on an earlier data extract that is at most 3 h old. In the rare case where errors would affect end-users, we developed email templates to: (1) notify end-users of the unplanned downtime; and (2) notify them when the unplanned downtime was over.

Planned downtimes are inevitable (e.g., due to database updates, server updates) and we also developed email templates accordingly.

### End-user engagement

End-users were engaged through the full life-cycle of this project as described in the methods and previous manuscripts (2, 10). A comprehensive effort was made to train physicians and nurses prior to, and during the deployment of CHARTwatch about the system, the interpretation of risk groups, the meaning of alerts, and the expected clinical responses. We incorporated CHARTwatch training into the orientation of all new nurses, such that all nurses working on the GIM ward receive CHARTwatch training. CHARTwatch training was integrated into the monthly orientation for residents, which includes in-person and emailed materials, and involves approximately 100 resident physicians annually. GIM staff physicians were trained through several presentations at division meetings and all GIM physicians (approximately 20) received the training.

### Performance measures

To measure model performance, we report the area under the receiver-operative curve (AUC), the positive prediction value (PPV), and sensitivity. We compute these metrics in the heldout test data (January 2020–May 2020), and in the real-time data from deployment (August 2020–March 2022). The model PPV is computed at the encounter-level. We calculate sensitivity based on the visit's maximum risk group, in order to get an estimate of the proportion of outcomes that would be captured by the visit's maximum risk group. We want this metric to be low for patients whose maximum risk group is “Low risk”.

To estimate adherence to clinical pathways, we used the number of vital sign assessments in the 24 h following an alert. This reflects both physician and nurse adherence as physicians must place an order and nurses must perform the measurements. According to the clinical pathway, vital signs should be measured every 4 h (the maximum frequency of routine measurement for patients on the GIM ward). Thus, adherence is measured as the total number of alarms that follow the clinical pathway (i.e., are followed by vital signs measurements every four hours) divided by the total number of alarms. We compute this metric at a weekly level and report the weekly percentage of alerts that follow the clinical pathway.

## Results

### Model performance

Model performance metrics on the heldout test data (January 2020 to May 2020, silent testing period) and the deployment data (August 2020 to March 2022) are reported in Table 2. When predicting the composite outcome of ICU transfer and in-hospital mortality, the time-aware MARS model achieved an AUC of 0.786 and of 0.759 on the test data and deployment data, respectively. When predicting patient deterioration within the next 48 h, the AUC was of 0.626 and 0.753.

**Table 2:** Performance of the CHARTwatch model in the test data and the deployment data. AUC, PPV, and sensitivity are reported in the test data (January 2020–May 2020) and in the deployment data (August 2020–March 2022). Metrics are reported on the composite outcome of ICU transfer and in-hospital mortality (Outcome: ICU/Death), as well as in the composite outcome of ICU transfer, in-hospital mortality, step-up unit transfer, and Palliative Care transfer (Outcome: ICU/Death/step-up/Palliative).

Metric	Test Data		Deployment Data	
	Outcome: ICU/Death	Outcome: ICU/Death/step-up/Palliative	Outcome: ICU/Death	Outcome: ICU/Death/step-up/Palliative
AUC (ever)	0.786	0.735	0.759	0.768
AUC (in next 48 h)	0.626	0.791	0.753	0.759
PPV of alerted encounters	0.172	0.306	0.257	0.272
Sensitivity (based on maximum risk group)				
High risk	0.480	0.53	0.565	0.559
Medium risk	0.520	0.471	0.419	0.417
Low risk	0	0	0.016	0.023

Maximum risk group sensitivity was of 0.530 for the “High risk” group, 0.471 for the “Medium risk” group, and 0 for the “Low risk” group in the test data. In production, the risk group sensitivity was of 0.559, 0.417, and 0.023.

During validation, we iterated through a range of risk score values and selected a threshold that would yield a PPV of 0.40 on the composite outcome of ICU transfer, in-hospital mortality, step-up unit transfer, and Palliative Care transfer. With this outcome, the model achieved a PPV of 0.306 and 0.272 in the test data and deployment data, respectively. In the composite outcome of ICU transfer and in-hospital mortality only, the model achieved a PPV of 0.172 and 0.257 in the test and deployment data, respectively.

### Alerts

**Figure 1:**
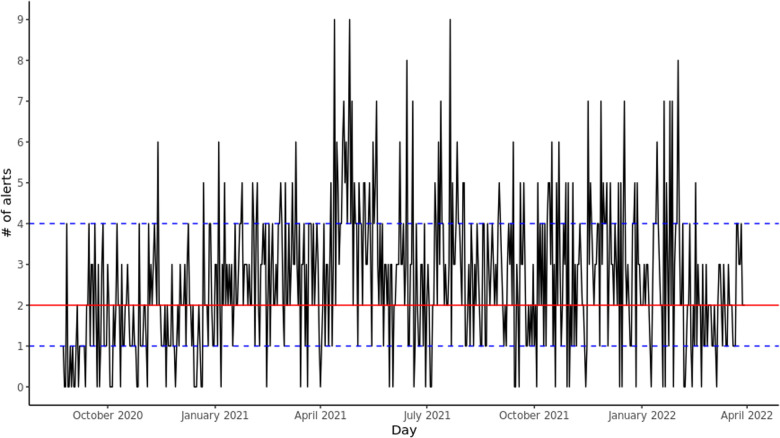
Daily alerts sent by CHARTwatch. The red solid line indicates the median number of daily alerts. The blue dashed lines indicate the 25th quantile and the 75th quantile.

Since deployment, there has been a mean of 2.60 (SD: 1.71) alerts per day and a median of 2 (IQR: [1–4]), for a mean of 84.02 (SD: 7.48) and median 84 (IQR: [79–89]) total GIM patients per day. There were 56 (9.589%) days where no alerts were sent. Figure 1 shows the daily number of alerts since deployment. The alerts were equally spread out across the different GIM teams.

### Adherence to clinical pathway

To assess adherence, we reported the percentage of alerted patients who had at least 4 vital signs measurements. The weekly percentage of adherence increased as users became more familiar with the system. Between August 2020 and November 2020, this weekly percentage was at a mean of 65% (SD: 11%) and a median of 65% (IQR: [59%–75%]). Between December 2021 and March 2022, this weekly rate had increased to a mean of 74% (SD: 11%) and a median of 71% (IQR: [69%–80%]).

### CHARTwatch pipeline runtime and data size

The pipeline runtime remains consistent and takes a mean of 196.83 seconds (SD: 121.05 seconds) to complete and a median of 151 seconds (IQR: [133–194] seconds). Similarly, the data size remains consistent at a mean of 26.44 MB (SD: 3.98 MB) and a median of 26.25 MB (IQR: [24.42–28.06] MB).

### Downtime events

We experienced few downtime events and most of them were planned. In total, the system was down for 52.5 out of 14,016 h (584 days). Thus, CHARTwatch was running for 99.6% of the time. 20 h (38.1%) of downtime were planned (scheduled database maintenance/upgrade, updates to server, etc.), and 32.5 h (61.9%) of downtime were due to unplanned events (such as unexpected database or network failure).

### Adherence to GMLP guidelines

1. **Multi-Disciplinary Expertise Is Leveraged Throughout the Total Product Life Cycle**. CHARTwatch was developed and deployed by a team from various fields of expertise with strong end-user engagement including advice from patients and caregivers, as previously described in detail (2).
2. **Good Software Engineering and Security Practices Are Implemented**. Our infrastructure follows best practices for security; the deployment server for CHARTWatch sits in the same secure private network as the clinical systems. Access to all systems is restricted to authorized personnel and continuously audited. Database administrators of clinical systems provided guidance to data engineers in developing high performance queries. The data pipelines were coded using techniques to minimize the risk of SQL injection in case of a system breach, while leaving a minimal footprint on the source systems. Furthermore, the data science team employed an agile development approach to develop the final deployed product. This included regular meetings to assess tasks, re-visiting the backlog and prioritizing as needed.
3. **Clinical Study Participants and Data Sets Are Representative of the Intended Patient Population**. The model was trained on historical hospitalizations from the same patient population at the same institution, to maximize representativeness. During model development, we worked directly with source database systems to ensure high quality data, including performing clinical validation ensured that the data sets were representative of real-world data.
4. **Training Data Sets Are Independent of Test Sets.** Our training data sets and test data sets were independent of each other. We used calendar-based data split approach to ensure that performance reported on the test set would be representative of deployment-level performance by simulating historical training and deployment in a future time period. Furthermore, silent testing did not overlap with our training/validation/test datasets.
5. **Selected Reference Datasets Are Based Upon Best Available Methods.** We tried multiple models and settled on the one that gave us the best performance and could work within constraints and limitations set by the system. Further, the features and models used in CHARTwatch were backed by previous evidence (10, 11).
6. **Model Design Is Tailored to the Available Data and Reflects the Intended Use of the Device**. When developing the model, we used data available at time of each prediction. Any data generated or updated after the expected prediction time was excluded from the training dataset. The silent deployment periods also allowed us to validate this.
7. **Focus Is Placed on the Performance of the Human-AI Team.** The clinical team was involved in development and deployment with regular meetings with all stakeholders and with extensive training on how to use the system. We suggested CHARTwatch predictions be used by clinicians in conjunction with their own clinical judgement rather than in isolation. Further, we conducted a clinical validation, comparing CHARTwatch model predictions to more than 3,000 real-time clinical predictions, to engender trust and inform our understanding of the human-AI team (manuscript under review).
8. **Testing Demonstrates Device Performance during Clinically Relevant Conditions**. We had silent testing periods as well as a pilot phase and a phased rollout. Model performance was monitored throughout the silent testing period and continues to be monitored on an ongoing basis.
9. **Users Are Provided Clear, Essential Information**. To ensure the delivery of simple, actionable messages for clinicians, CHARTwatch predictions were categorized into “High risk”, “Medium risk”, and “Low risk” groups. Messaging alerts contain the following text: “[Patient Last Name, First Name, Medical Record Number] is high risk for transfer to ICU or death. Please refer to **LINK** for more information.” The link takes users to a brief description of the clinical protocol for alerted patients. All clinicians receive training on how to use this system.
10. **Deployed Models Are Monitored for Performance and Re-training Risks are Managed.** Model performance is measured and monitored by an implementation committee, using a small number of key performance and process measures, including those reported in this manuscript: model sensitivity, PPV, number of daily alerts, number of outcomes for patients in different risk groups, and number of vital signs measurements in the 24 h following an alert. These were initially monitored weekly and once the intervention moved into a more stable maintenance phase, committee meetings are held monthly. Error alerts on the automated pipelines ensure timely identification of errors by the team. Re-training poses an important challenge, as the model has altered clinical workflows, particularly for alerted patients. Given that clinical interventions are intended to prevent adverse outcomes for alerted patients, re-training the model may lead to undesired feedback loops resulting in poorer performance. This remains an area of active research for our team and others (12), including exploring the use of proxy labels to ensure that high risk patients who do not experience adverse outcomes are still captured in the modelling.

## Discussion

In this manuscript, we describe our experience deploying an early warning system for GIM patients in an academic hospital, which highlights numerous practical lessons. We observed that the GMLP guiding principles offer a helpful starting point, and our solution was developed in alignment with these suggestions. We offer concrete and detailed descriptions of how we were able to operationalize the various GMLP recommendations, to assist future initiatives. Beyond these principles, we identified several aspects that have been critical for successful deployment of our solution. First, engagement of end-users was essential in designing, deploying, and iteratively refining the solution. Second, a silent testing period and phased launch was crucial for identifying unanticipated issues with models and data pipelines and resulted in numerous updates before launch. Third, it was important to create robust downtime protocols, with a careful plan to prevent disruptions in clinical workflow or patient harm.

Engaging a multidisciplinary group of end-users from the project's outset ensured that there was a high level of trust and uptake of the designed solution. We discuss in more detail our findings of engaging a multidisciplinary group in (2). Ongoing engagement led to important iterations in the intervention. Our engagement included identifying key champions to participate in committees and lead the initiative and a comprehensive training program for all clinicians. Regular implementation committee meetings, initially held weekly and then scaled back over time to monthly, allowed the team to refine the intervention in response to feedback from clinicians.

We achieve high model performance and, on average, CHARTwatch only sends out two alerts each day. The outcomes not captured by the alert are captured by the “Medium risk” group. Combining the “High risk” and “Medium risk” groups together yields a sensitivity of 0.976. In addition to monitoring various measures of model performance and clinical outcomes, we used a simple process measure to capture clinical adherence (the number of vital signs measurements). This reflects both physician and nurse practice and demonstrated good adherence at the project's outset, with further improvements over time.

There is a notable absence of “best practices” in deploying ML solutions in healthcare. The GMLP guiding principles are an important step forward and as high-level guides, they are very useful. However, greater specificity is needed to understand how these principles can be operationalized, and this manuscript reflects an effort to provide some of that additional detail. We also note that there are several crucial areas for ML deployment which are referred to only tangentially in the GMLP and ought to be mentioned specifically. GMLP Principle 1 (“Multi-Disciplinary Expertise Is Leveraged Throughout the Total Product Life Cycle”) is very applicable to the importance of end-user engagement, although it does not mention this specifically. GMLP Principle 8 (“Testing Demonstrates Device Performance during Clinically Relevant Conditions”) may be strengthened by highlighting the importance of silent testing in a real-time production environment before deployment into clinical care. An important area for future research is to develop a guiding framework that would help determine what duration of silent testing is sufficient before deployment. This duration would be affected by parameters related to model performance (e.g., prevalence of outcome events, desired model accuracy) and factors related to the data pipeline and clinical context (e.g., number of clinical systems involved, planned updates to systems and processes). GMLP Principles 9 (“Users Are Provided Clear, Essential Information”) and 10 (“Deployed Models Are Monitored for Performance and Re-training Risks are Managed”) should be expanded to include downtime protocols. While system and model failures are rare, they are bound to happen, and end-users should not be left in the dark. Borrowing from safety engineering, failure modes and effect analysis could be a good way to identify all potential risks within the deployed system and, accordingly, develop downtime protocols (13). Finally, we note that GMLP Principle 10 raises a crucial area for future research. Re-training models that have already been deployed into, and affected, clinical practice raises challenging methodological issues (12). Identifying methods to maintain highly accurate models over time is an urgent need as models are increasingly deployed into clinical environments.

This work has several limitations. First, our deployment was conducted in a single academic hospital and thus generalizability to other settings must be considered. However, we believe the key lessons from our experience are very likely to apply to a wide range of ML solutions. Second, our deployment relates to a clinical decision-support and predictive analytics solution. Other ML applications (e.g., computer vision) may require a different set of approaches for their deployment. Third, we relied primarily on routinely-collected data to measure model performance and clinical adherence. This has the advantage of being scalable and resource-efficient, but lacks granularity and clinical context. Targeted chart reviews, as have been described in the quality improvement literature (14), or interviews with clinicians represent other important ways of gathering this information.

In conclusion, deploying machine learning models in healthcare settings is challenging and requires a multi-disciplinary team to ensure success. As these deployments become more frequent, we hope that more rigorous standards and best practices will arise. The evolution of the GMLP guiding principles, and lessons learned from real-world implementations, can assist with strengthening best practices in the deployment of machine learning models.

## Data Availability

The data is not publicly available as it contains personal health information (PHI).

## Footnotes

^1^ https://www.spok.com/

## Appendix Table 1: Table of scheduled scripts.

**Table:**

Script	Description	Schedule
CHARTwatch pipeline	The script extracts data from the source systems, processes the data, generates model prediction, and classifies each patient into risk groups (“Low risk”, “Medium risk”, “High risk”).	Hourly
Charge nurse email	The scripts sends a list of the patient census, including CHARTwatch risk groups, to the charge nurse twice a day.	Every 12 h
SPOK alert update	The script sends alerts to the “SPOK” application on the GIM team phones and charge nurse phone. The script sends alerts on “High risk” patients and applies re-alerting silencing rules (as specified in Section “Description of system”).	Hourly
“Electronic sign out” tool update	The script updates the CHARTwatch risk groups in the “electronic sign out” database.	Hourly
Palliative team email	The script sends a daily email to the Palliative care team. The email contains a list of all new “High risk” patients.	Daily

## Appendix Table 2: Timeline of CHARTwatch major changes

**Table:**

Date	Change	Type of change
13-Nov-19	**Silent deployment**. Deployment was on an older server and consisted in an ensemble model that used the following input data: demographics, labs, vitals, medications, clinical orders, nursing notes.	Silent deployment
26-Nov-19	New “high sensitivity troponin” lab added.	Process change
20-Dec-19	**Silent deployment update.** The model was updated to an ensemble that relied only on demographics, labs, and vitals. We also fixed an issue where sodium labs (lab code is “NA”) were getting interpreted as “Not available”.	Silent deployment
24-Dec-19	**Silent deployment update.** The data processing was changed to address the troponin lab update.	Silent deployment
14-May-20	**Silent deployment update**. The deployment was moved to a different server and we used the time-aware MARS model (as described in Section “Model development”).	Silent deployment
25-Aug-20	**Start of pilot phase.** Alerts and ‘electronic sign out' was activated for 2 GIM teams.	Deployment
11-Sep-20	Risk group rule change: if patient is on step-up unit, their risk group must at minimum be “Medium risk”.	CW change
11-Sep-20	Alerting rule change: Alerts silenced for 24 h after patient leaves ICU.	CW change
11-Sep-20	Add “Team Stroke” to data extraction query.	Process change
11-Sep-20	Add new GIM ward location to data extraction query, corresponding to the opening of a new patient care tower.	Process change
15-Sep-20	**Launch to all GIM teams.**	Deployment
6-Oct-20	**Deployment to charge nurses.** Emails are sent to the charge nurse email address twice a day. Alerts are sent to the charge nurse phone.	Deployment
20-Oct-20	**Deployment to palliative team—Full deployment.** Emails are sent daily to the Palliative Care team email.	Deployment
19-Jan-21	Switch from alerts 3×/day to hourly alerts.	CW change
27-Apr-21	Add an extra GIM team (opened for COVID-19) to data extraction query.	Deployment
11-Jun-21	Remove extra COVID-19 team from data extraction query, as team closed.	Deployment
8-Mar-22	Alerting rule change: stop repeat alerts after 5th alert.	CW change

## Appendix 3: Details on the CHARTwatch model

The final deployed model consists of two pieces:

(1) The Multivariate Adaptive Regression Spline (MARS) is a weighted sum of basis functions (15). To determine the coefficients, we used cross-validation to train the model. The inputs to the MARS model are the features described in Section “Model development”.
(2) The output of the MARS model is a score ranging from 0 to 1. The MARS score, as well as the difference in MARS score between this time window and the previous one, the percent change, and the percent change from baseline, are then given as input to a logistic regression model. The logistic model is trained on the training data.

The MARS and logistic regression models were trained using R (version 3.6.3) (16) with the packages ‘earth’ (version 5.1.2) (17) and ‘glmnet’ (4.0-2), respectively (18). We use the ‘tidymodels’ suite of packages (19) to train and tune the models.
